# Neural alterations in opioid-exposed infants revealed by edge-centric brain functional networks

**DOI:** 10.1093/braincomms/fcac112

**Published:** 2022-05-05

**Authors:** Weixiong Jiang, Stephanie L Merhar, Zhuohao Zeng, Ziliang Zhu, Weiyan Yin, Zhen Zhou, Li Wang, Lili He, Jennifer Vannest, Weili Lin

**Affiliations:** 1 Biomedical Research Imaging Center, University of North Carolina at Chapel Hill, Chapel Hill, NC, USA; 2 Perinatal Institute, Division of Neonatology, Cincinnati Children’s Hospital and University of Cincinnati Department of Pediatrics, Cincinnati OH, USA; 3 East Chapel Hill High School, Chapel Hill, NC, USA; 4 Department of Biostatistics, University of North Carolina at Chapel Hill, Chapel Hill, NC, USA; 5 Department of Radiology, Cincinnati Children’s Hospital and University of Cincinnati, Cincinnati OH, USA; 6 Department of Communication Sciences and Disorders, University of Cincinnati, Cincinnati OH, USA; 7 Department of Radiology, University of North Carolina at Chapel Hill, Chapel Hill, NC, USA

**Keywords:** prenatal opioid exposure, functional MRI, dynamic functional connectivity, brain network, edge-centric functional networks

## Abstract

Prenatal opioid exposure has been linked to adverse effects spanning multiple neurodevelopmental domains, including cognition, motor development, attention, and vision. However, the neural basis of these abnormalities is largely unknown. A total of 49 infants, including 21 opioid-exposed and 28 controls, were enrolled and underwent MRI (43 ± 6 days old) after birth, including resting state functional MRI. Edge-centric functional networks based on dynamic functional connections were constructed, and machine-learning methods were employed to identify neural features distinguishing opioid-exposed infants from unexposed controls. An accuracy of 73.6% (sensitivity 76.25% and specificity 69.33%) was achieved using 10 times 10-fold cross-validation, which substantially outperformed those obtained using conventional static functional connections (accuracy 56.9%). More importantly, we identified that prenatal opioid exposure preferentially affects inter- rather than intra-network dynamic functional connections, particularly with the visual, subcortical, and default mode networks. Consistent results at the brain regional and connection levels were also observed, where the brain regions and connections associated with visual and higher order cognitive functions played pivotal roles in distinguishing opioid-exposed infants from controls. Our findings support the clinical phenotype of infants exposed to opioids in utero and may potentially explain the higher rates of visual and emotional problems observed in this population. Finally, our findings suggested that edge-centric networks could better capture the neural differences between opioid-exposed infants and controls by abstracting the intrinsic co-fluctuation along edges, which may provide a promising tool for future studies focusing on investigating the effects of prenatal opioid exposure on neurodevelopment.

## Teaser

Jiang *et al* reported brain regions, connections, and networks capable of distinguishing opioid-exposed infants from controls using dynamic functional connectivity (dFC). In particular, prenatal opioid exposure appears to preferentially affect inter- instead of intra-network dFC in relation to visual, subcortical, and default mode networks.

## Graphical Abstract

**Graphical Abstract:**
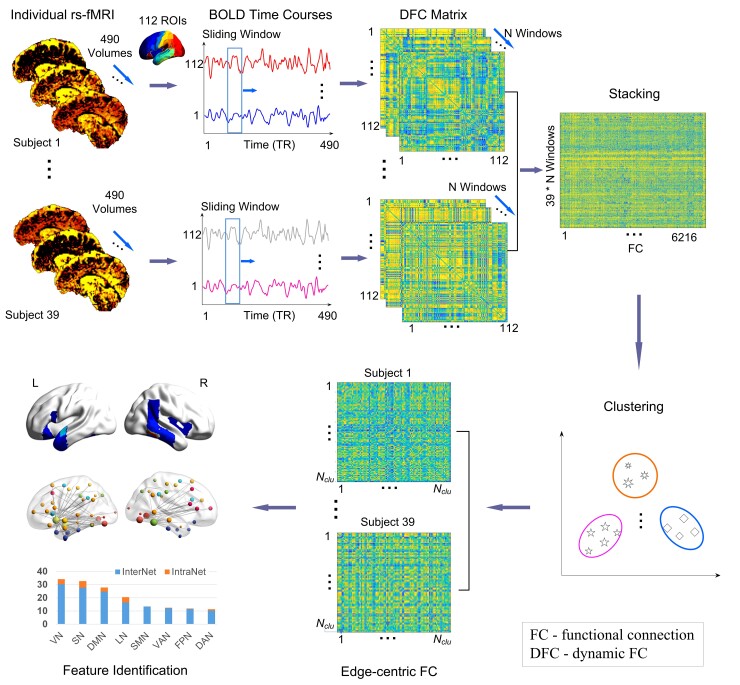

## Introduction

Opioid use disorder (OUD) can lead to devastating health problems in adults, including impacts on brain structure and function. Structural and functional MRI in adult patients with OUD have revealed decreased brain volumes in the frontal and temporal lobes, functional abnormalities of the prefrontal cortex and temporal lobe,^1^ and altered functional connectivity (FC) patterns at both the network- (reward and executive control systems) and regional-level (prefrontal cortex, amygdala, orbitofrontal cortex, cingulate cortex, hippocampus, and thalamus).^2–4^ These aforementioned studies have provided valuable insights into how brain structure and function may be altered in adults with long-term exposure to opioids. However, effects of opioid exposure on the developing brain in utero are less well-understood. Clinical manifestations of prenatal opioid exposure (POE) are well-documented; in particular, the opioid epidemic has yielded a ∼five-fold increase in the incidence of neonatal opioid withdrawal syndrome.^5^ Later in development, prenatally opioid-exposed children differ from unexposed children in multiple neurodevelopmental domains, including cognition, motor development, attention, and vision.^6–11^ However, alterations in brain structure and function that could underlie these developmental consequences are just beginning to be investigated. Preclinical animal studies suggest a possible causal relationship between POE and subsequent negative outcomes, including decreased neurotransmitter levels,^12–14^ decreased neurogenesis,^15,16^ increased apoptosis,^17^ and altered myelination^18,19^ in opioid-exposed rodents compared with controls. In addition, impaired learning and memory has been found in murine offspring exposed to opioids prenatally.^20–23^

In order to investigate the neural underpinnings of altered neurodevelopment in infants with POE, non-invasive imaging approaches, particularly MRI, have been used. Altered brain microstructural and structural features in POE infants using MRI have been reported, including decreased fractional anisotropy in the major white matter tracts,^24^ and altered volumes in multiple brain regions when compared to controls.^25^ Furthermore, the use of resting state functional MRI (rs-fMRI) has enabled the investigation of altered FCs and networks resulting from prenatal drug exposure.^26,27^ Nevertheless, there have been only two prior studies reporting altered FCs in prenatal opioid-exposed infants. We recently reported that the brains of POE infants exhibited smaller network volumes, particularly in the primary visual network, and different network topologies from controls, particularly in visual and executive control networks.^28^ In addition, Radhakrishnan et al.^29^ reported significant differences in connectivity between an amygdala seed to several cortical regions in POE infants. While these studies revealed potential neural FC alterations in POE infants, they are largely based on a static FC matrix calculated using the entire rs-fMRI scan.^26–29^ Static FCs are believed to reflect ‘core connections’ of the brain functional networks, but do not account for adaptive ‘dynamic’ brain functional organization.^30,31^ Alternatively, dynamic FC (dFC) methods reflect intrinsic brain properties with a neural origin and capture subtle changes in neural activity patterns underlying cognition and behaviour.^30,32–34^ In particular, based on dFC, a large-scale edge-centric network, which has also been referred to as a high-order network to distinguish it from traditional static FC networks, has led to a higher sensitivity (SEN) for detecting disease-related functional abnormalities when compared to the traditional static FC.^35–37^

In this study, we aimed to (i) differentiate POE infants from unexposed controls using an edge-centric high-order FC classifier and (ii) extract POE-related neural features associated with canonical networks supporting sensory and cognitive functions that may be affected by POE. We hypothesized that the edge-centric high-order network patterns would improve our ability to differentiate infants with POE from controls when compared to static FCs, and could potentially reveal the underlying neural abnormalities associated with POE infants at regional, connection, and network levels.

## Materials and methods

A total of 49 infants, including 21 prenatal opioid-exposed and 28 controls born at ≥ 37 weeks gestation without other medical problems were recruited from Cincinnati Children’s Hospital, birth hospitals in the Greater Cincinnati area, or through community research advertisements. POE status was determined by maternal history and/or maternal urine toxicology screen at the time of delivery and was further confirmed with neonatal toxicology screen (meconium or umbilical cord). Lack of POE in controls was also confirmed by negative maternal urine toxicology screens at time of delivery (done universally in our area birth hospitals) as well as history. Additional information including drug exposure, maternal socioeconomic status (including education, employment, and income) and race, pregnancy and birth history, was collected by review of infant medical records or/and by maternal questionnaire at the time of MRI. This study was approved by the Institutional Review Boards at Cincinnati Children’s Hospital, Good Samaritan Hospital, and St. Elizabeth Hospital. Written informed consent was obtained from a parent or guardian prior to any study procedures.

### MRI imaging acquisition and preprocessing

All infants were scanned during sleep without sedation on a Philips 3 T Ingenia scanner with a 32-channel receive head coil in the Imaging Research Center at Cincinnati Children’s Hospital. Infants were placed in the Med-Vac vacuum bag (CFI Medical Solutions, Fenton MI) with ear protection when in the scanner. Resting state fMRI was acquired using an axial gradient echo-planar imaging sequence with simultaneous multi-slice excitation (multi-band). Imaging parameters were as follows: repetition time (TR) = 1011 ms; echo time (TE) = 45 ms; flip angle (FA) = 54°; resolution = 2.25 × 2.25 × 2.25 mm^3^; 60 contiguous slices; multi-band factor = 6; 500 time-series volumes and scan time 8 min 37 s. Structural MRI sequences included a sagittal magnetization prepared inversion recovery 3D T1-weighted gradient echo sequence (shot interval = 2300 ms, RE = 7.6 ms, TE = 3.6 ms, inversion time = 1100 ms, FA = 11 degrees, resolution = 1 × 1 × 1 mm^3^, acceleration (sensitivity encoding (SENSE) = 1 in plane and 2.0 through plane (slice) phase encode, scan time 3 min 6 s) and an axial 2D T2-weighted fast spin echo sequence (TR = 19 100–19 500 ms, TE = 166 ms, resolution = 1 × 1 × 1 mm^3^, acceleration (SENSE) = 1.5 in plane phase encode, scan time 3 min 50 s). A board-certified paediatric neuroradiologist reviewed all structural images to confirm no clinically relevant abnormalities.

An in-house infant-specific fMRI pipeline was used to preprocess rs-fMRI data.^38–40^ This pipeline shares some common steps with the Human Connectome Project (HCP) pipeline (https://github.com/Washington-University/Pipelines), including head motion correction, aligning rs-fMRI images to T1-weighted image space, and band-pass filtering (0.01–0.08 Hz) and several additional unique steps tailored to infant FC MRI.^41^ Brain tissue segmentation was first conducted to generate tissue labelling maps (each voxel was assigned as grey matter, white matter, or cerebrospinal fluid) using a multi-site infant-dedicated computational toolbox, iBEAT V2.0 Cloud (http://www.ibeat.cloud).^42^ The tissue labelling maps were used to register to the Montreal Neurological Institute (MNI) template space (ICBM_152_t1_tal_nlin_sym09c, without cerebellum), minimizing the registration difficulties associated with age-dependent signal intensity and low tissue contrast in anatomical images.^38,39^ Furthermore, using an independent component analysis (ICA) approach to derive independent components, unsupervised deep-learning methods were performed to detect and regress noise-related components.^41^ All preprocessing steps, including resampling, band-pass filtering, and denoising, were conducted in each subject’s native space. The subjects with a mean Power framewise displacement (FD) larger than 0.5 mm were excluded.^43^

### Constructing eFC

**Figure 1:**
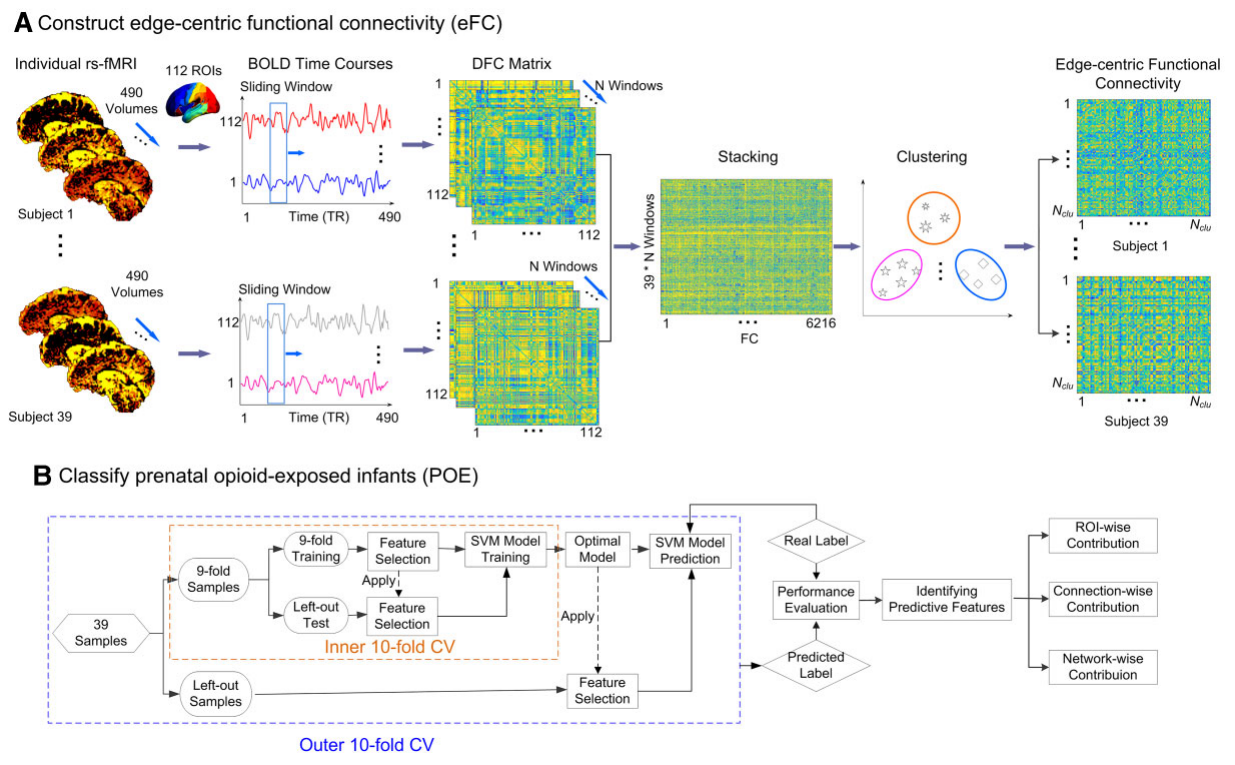
**Schematic of eFC-based classification and discriminative feature identification for prenatal opioid-exposed infants.** (**A**) The construction of eFC and (**B**) the classification of prenatal opioid-exposed infants and the identification of discriminative features with nested 10-fold CV which consisted of inner and outer layers. The inner 10-fold CV was used to optimize the predictive model by feature selection and the optimal model was used to generate the results of the left-out samples in an outer 10-fold CV.

Fig. 1A shows the steps to construct edge-centric FC (eFC) matrices.^36^ The preprocessed functional data was parcellated into 112 regions of interest (ROIs, Supplementary Table 1) using the Harvard–Oxford atlas. The atlas in the standard MNI space was warped to each subject’s native space to extract regional averaged blood oxygenation level dependent (BOLD) signals. A sliding window approach using window lengths ranging between 50 and 120 TRs with a 10-TR increment and a step size of 1 TR for the sliding window was employed. An FC matrix was calculated for each sliding window using Pearson’s correlation among pairs of ROI signals. The lower triangle of the FC matrix (112 × (112–1)/2 = 6216 edges) was extracted from each window. By concatenating all sliding windows, a vector with a length of *6216 × N_window_* where *N_window_* is the number of windows obtained for each subject. To reduce the eFC dimension, we further concatenated edge time series across all subjects along the windows [6216 × (*N_window_ × N_subjects_*)] and a clustering approach was employed to group synchronized edge time series into clusters.

Using this method, the edges assigned to the same cluster possessed a similar temporal pattern of variation across all subjects. Subsequently, the mean cluster time series (*N_cluster_ × N_window_*) of each subject was calculated by averaging the edges assigned to the same cluster. Finally, eFC (*N_cluster_ × N_cluster_*) of each subject was obtained by calculating the Pearson’s correlation of different averaged cluster time series. In an eFC network, each node represents an edge-centric cluster involved in highly synchronized dFCs, while each edge represents the interaction between each pair of clusters, reflecting a high-order brain functional organization. Of note, in order to obtain optimal classification, we adopted a series of window sizes from 50–120 TRs with a step of 10 TRs, and a series of clusters from 100–800 clusters with a step of 100. Additional details are provided in the next sub-section.

### Differentiating POE infants from normal controls

We developed a nested 10-fold cross-validation (CV) support vector machine (SVM) using least absolute shrinkage and selection operator (LASSO) feature selection to differentiate POE from controls (Fig. 1B) using eFC. The weighted-graph local clustering coefficients, which quantified high-order local connectivity or ‘cliqueness’ of a given eFC network,^44,45^ were obtained by computing the probability of the neighbours of a given vertex that are also connected to each other. The weighted-graph local clustering coefficients were used to extract features from each e FC network.^44,45^ For each cluster, we obtained one value presented as the weighted-graph local clustering coefficient. We chose this measure to decrease the feature dimensions from *N_cluster_* × *N_cluster_* to *N_cluster_* while retaining the local connectivity characteristics. These local clustering coefficients were used as inputs of the following classifier.

To determine the optimal model for differentiating POE from controls, nested 10-fold CV was employed. For each outer 10-fold CV, the dataset was randomly separated into 10 subsets where nine subsets were used for training while the remaining one dataset was used for testing. The above procedures were repeated 10 times and the mean area under the curve (AUC) of receiver operating characteristics, accuracy (ACC), SEN, and specificity (SPE) were used to determine the performance of the model.^46^

The classification ACC was related to the window lengths and number of clusters (Supplementary Fig. 1). A representative example of the eFC differences between POE infants and controls using a window length of 50 TRs and a step size of 10 TRs were showed in the supplement materials (Supplementary Table 2). To optimize the combination of the window lengths (window length: 50–120 TRs and a step size: 10 TRs) and number of clusters (100–800 clusters with a step of 100), we used the inner 10-fold CV methods. The model that yielded the best classification performance (the largest ACC) with 10-fold CV was chosen as the optimal model to predict the test samples in the outer 10-fold CV (Fig. 1B). For each inner 10-fold CV, we used the LASSO method to select the crucial eFC features under each combination of sliding window and cluster of the training datasets, so as to reduce possible feature redundancy and improve the classification performance and model robustness.^47^ The selected features were used to train the SVM model that was then used to predict the testing samples of the inner 10-fold CV.

In this study, a soft-margin SVM was chosen to classify high-dimensional data, which identified the maximal margin between two groups while allowing some misclassifications.^48^ Such a soft margin method has been well-validated, extensively used and demonstrated superior performance even with a small sample size.^34,35,49^ Herein, we used a linear kernel with a hyperparameter *C* = 1^34,50,51^ and other hyperparameters were kept as defaults to make the model more robust,^35^ including *insensitivity* = 0 and an *eInsensitive* loss function.^34,50,51^

### Identification of POE specific neural substrates

In order to identify specific neural substrates capable of distinguishing POE from controls, a tracing back method was employed based on the eFC classifier to derive quantitative features from three levels, namely connection-wise, region-wise, and network-wise contributions such that the potential POE-related neural alterations at the FC, regional, and network levels could be discerned. Specifically, in the linear SVM, a hyperplane was created by using support vectors to maximize the distance between the two classes. The obtained weights represent the vector coordinates orthogonal to the hyperplane. The absolute value of their coefficients in relation to each other can be used to determine feature importance for the data classification task.^52^ Based on the absolute coefficients of feature importance in linear SVM, we derived quantitative effects of POE at the aforementioned three levels.

#### Connection-wise contributions

The normalized weight of each cluster was calculated to assess the discriminative ability of distinguishing POE infants from controls. Specifically, throughout the entire classifying processes, i.e. 10 times 10-fold CV, the number of appearances of a given cluster was recorded. The occurrence rate was then calculated as the number of appearances of a given cluster divided by the total classifying number 100 (10 × 10 for 10 times 10-fold CV). Subsequently, the normalized weight was defined as the average SVM weight multiplied by the occurrence rate of each cluster across the outer 10-fold CV with 10 repetitions, Fig. 1B (i.e. the average SVM weight × the frequency of each cluster/100). The greater the normalized weight of a given cluster is, the stronger this cluster contributes to discriminate the two groups.^36^ Since each cluster involved many low-order or basic FCs, we traced back and summed up all normalized cluster-weights related to each basic FC (edge), reflecting the ‘connection-wise contribution’.

#### Brain region-wise contributions

Based on the low-order FC’s weights, we determined the weight of each brain region (*ROI-wise contributions*) by summing the absolute values of the ‘connection-wise contributions’ across all FC links that were connected to a given region.^48^ In this way, the ROIs with higher weights were deemed to be more discriminative in distinguishing POE from controls.

#### Network-wise contributions

To decide the network affiliation of each ROI, eight canonical networks were adopted, including the seven networks proposed by Yeo *et al*^53^ and a subcortical network (SN) by Finn *et al*^51^ (See Supplementary Table 3). The seven cortical networks included the fronto-parietal (FPN), ventral attention (VAN), default mode (DMN), dorsal attention (DAN), sensorimotor (SMN), visual (VN), and limbic network (LN). Each ROI was mapped to the network atlas (both in MNI space) and the number of overlapping voxels between each ROI and each network was calculated. The network affiliation of each ROI was decided by the proportion of overlapping voxels over the total voxel number of this ROI (*N_overloaping_/N_total_*). Through this method, some regions may be involved in two or three networks in different proportions (See Supplementary Table 3).

Subsequently, we calculated network-level contributions through intra-network, inter-network, and pairwise inter-network POE-associated indices. The intra-network index was calculated as an absolute sum of the connection-wise contributions across all selected intra-network edges, while the inter-network index was calculated by the absolute sum of the connection-wise contributions across all edges connecting one network to all the other networks. If a dFC link connected two networks, its connection-wise contribution was divided by two for each network. To further investigate the contribution of pairwise networks to the POE—brain association, we calculated the sum of the connection-wise contribution between each pair of networks, respectively.

### Statistical analyses

Statistical analyses of demographic/clinical data were performed using IBM SPSS Statistics 23. Descriptive statistics for demographic variables were computed for the two groups (opioid-exposed and controls). Two-sided *t*-tests were conducted for the comparison of continuous variables and Fisher’s exact test for categorical variables between two groups. A *P*-value <0.05 was considered significant. In addition, two-sample *t*-tests were used for the group comparison of static FC strength between POE infants and controls with false discovery rate (FDR) for correcting multiple comparisons (*P* < 0.05) using MATLAB 2018b.

### Validation

Permutation tests were used to assess the statistical significance of the model’s discriminative ability.^49,50^ The classification labels of the data were randomly permuted 5000 times, and the same SVM procedures with 10 times 10-fold CV was then performed on every permuted set. We defined the ‘real’ ACC as the ACC obtained by the classifier trained on the real class labels. If the real ACC exceeded the 95% (*P* < 0.05) confidence interval of the classifier trained on randomly re-labelled class labels, the classifier was considered to reliably learn the relationship between the data and the labels.

To further determine the effectiveness of the eFC-based POE classification, the eFC-based results were compared to that obtained using the traditional static FC method. The main difference between the two approaches was that Pearson’s correlation coefficients were calculated using rs-fMRI signals among pairs of brain regions within the entire scanning period and were used as the features for static FC-based classification.^49^ We then tested the differences of static FC strengths between groups using a two-sample *t*-test (*P* < 0.05, FDR corrected). We also evaluated the ACC, SEN, SPE, as well as AUC when the clustering coefficients of the static FC network were used as features.

### Data availability statement

The data included in this study is a part of ongoing studies and thus cannot be made publicly available at the present time. Nevertheless, we have shared our codes for the analysis of edge-centric functional networks through a repository https://github.com/Windywom/Classification-based-on-Edge-centric-FC.

## Results

Of the 49 infants (21 opioid-exposed and 28 controls) with completed MRIs, 10 infants were excluded due to excessive motion (Power FD > 0.5 mm), leading to a final sample of 15 opioid-exposed and 24 control infants for data analyses. All subjects were born at ≥ 37 weeks gestation and were scanned at approximately 6 weeks (43 ± 6 days) after birth, minimizing the effects of the postnatal environment on brain connectivity. Maternal smoking, maternal Hepatitis C, and maternal education were significantly different between the POE and control groups (Table 1).

**Table 1:** Demographics of study population

	Opioid-exposed (*n* = 15)	Controls (*n* = 24)	*P* value
Male, *n* (%)	6 (40)	9 (37.5)	0.57
Gestational age at birth (weeks), mean (SD)	38.7 (0.9)	39.1 (0.8)	0.096
Birth weight (g), mean (SD)	3070 (275)	3190 (361)	0.277
Head circumference at birth (cm), mean (SD)	34.3 (1.2)	33.9 (1.3)	0.394
Postmenstrual age at scan (weeks), mean (SD)	44.8 (1.2)	45.2 (1.4)	0.419
Race/ethnicity, *n*			0.092
Non-Hispanic White	12	11	
Non-Hispanic Black	2	10	
Hispanic White	1	3	
Maternal smoking, *n* (%)	14 (93)	1 (4)	<0.001^a^
Any maternal alcohol use during pregnancy	1 (7)	1 (4)	1
Maternal Hepatitis C, *n* (%)	10 (67)	0 (0)	<0.001^a^
Maternal college degree, *n* (%)	2 (13)	16 (67)	0.002^a^
Maternal methadone, *n* (%)	5 (33)	n/a	n/a
Maternal buprenorphine, *n* (%)	9 (60)	n/a	n/a
Maternal heroin and/or fentanyl, *n* (%)	8 (53)	n/a	n/a
Other maternal illicit drug use	3 (20)	n/a	n/a
Neonatal abstinence syndrome requiring opioid treatment, *n* (%)	4 (27)	n/a	n/a

Two-sided *t*-test was used to compare continuous variables and Fisher's exact test was used to compare categorical variables.

^a^ Represents significant difference between two groups.

### Performance of eFC-based classifier

**Figure 2:**
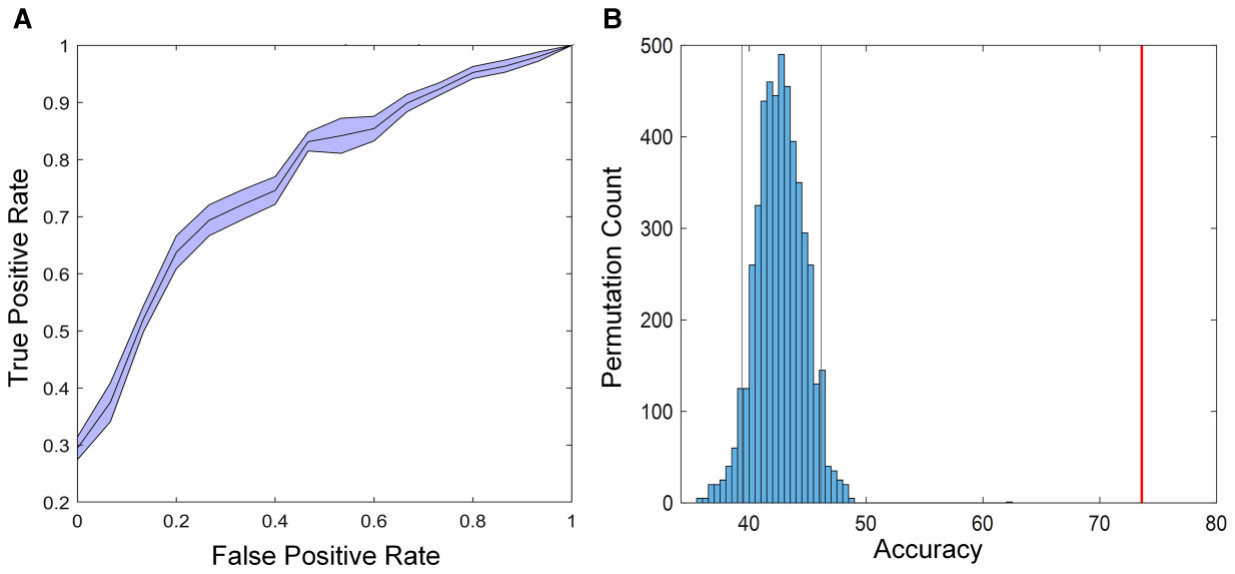
**The eFC-based classification performance.** (**A**) The AUC curve in the SVM classifier with 10 repetition 10-fold CV. (**B**) Permutation distribution of the estimate (TRs: 5000). Red line is the ACC obtained by the classifier trained on the real class labels based on the clinical assessments. Grey lines are the 95% (*P* < 0.05) confidence interval of the classifier trained on randomly re-labelled class labels. This figure presents that the classifier reliably learned the relationship between the data and the labels.

We hypothesized that dynamic rs-fMRI would outperform the widely used static rs-fMRI to distinguish POE infants from controls. The eFC achieves an average ACC of 73.6% (SEN: 76.25%, SPE: 69.33%, AUC: 0.7936, F1-score: 78.07) using 10 times nested 10-fold CV (Fig. 2A) which outperforms that obtained using the widely used static FC (ACC: 56.9%, sFC in Table 2) as well as combining clustering coefficients and the static FC (ACC: 51.96%, sFC + WLCC in Table 2), suggesting that dynamic rs-fMRI is more effective in identifying differences between POE infants and controls. The permutation tests showed that the proposed classifier reliably learned the relationship between the data and the labels, exceeding the 95% (*P* < 0.05) confidence interval of the classifier (Fig. 2B). Two-sample *t*-tests showed no significant differences of static FC strength between the POE infants and controls (*P* < 0.05, FDR corrected), which was consistent with the results in a recent study using the automated anatomical labelling (AAL) atlas (90 brain regions).^28^

**Table 2:** Comparisons of static versus dFC to distinguish POE infants from normal controls

Method	ACC (%)	SPE (%)	SEN (%)	AUC	F1-score (%)
sFC	56.92	66.67	41.33	0.5181	65.54
sFC + WLCC	51.96	67.92	36.00	0.5425	65.28
eFC	73.59	76.25	69.33	0.7936	78.07

sFC, static functional connectivity; WLCC, weighted local clustering coefficients.

### Region-wise contributions distinguishing POE infants from controls

**Figure 3:**
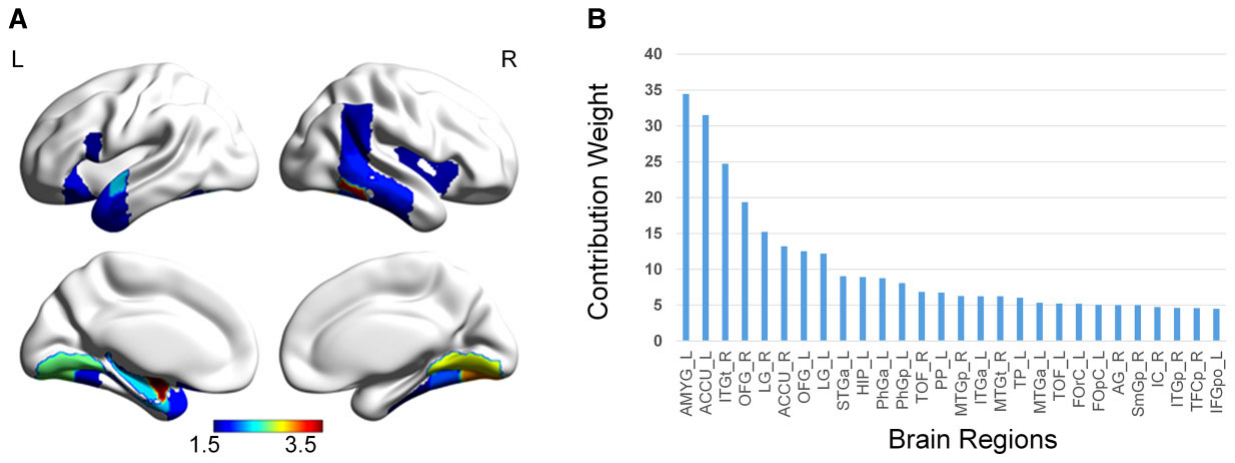
**Region-wise contributions distinguishing the opioid-exposed infants from controls.** (**A**) The surface view of the highest 25% ROIs. The colour bar shows normalized weights, reflecting the importance of ROIs in the classifier model (i.e. the degree of contribution). (**B**) The normalized weights of the highest 25% ROIs. AMYG, amygdala; AG, angular gyrus; ACCU, accumbens; ITGt, inferior temporal gyrus, temporooccipital part; FOrC, frontal orbital cortex; FOpC, frontal operculum cortex; HIP, hippocampus; IC, insular cortex; ITGp, inferior temporal gyrus, posterior division; PhGa, parahippocampal gyrus, anterior division; PhGp, parahippocampal gyrus, posterior division; TOF, temporal occipital fusiform cortex; PP, planum polare; MTGp, middle temporal gyrus, posterior division; ITGa, inferior temporal gyrus, anterior division; MTGt, middle temporal gyrus, temporooccipital part; MTGa, middle temporal gyrus, anterior division; STGa, superior temporal gyrus, anterior division; TP, temporal pole; SmGp: supramarginal gyrus, posterior division; TFCp, temporal fusiform cortex, posterior division; L, left; R, right.

The ROI-level weights, reflecting the discriminative ability of a given ROI for differentiating POE infants from controls, are provided in Supplementary Table 2 and the brain regions with the highest 25% of weights are shown in Fig. 3. These regions were mainly in the subcortical regions, visual regions, temporal lobe, and other high-order brain regions.

### Connection-wise contributions distinguishing POE infants from controls

**Figure 4:**
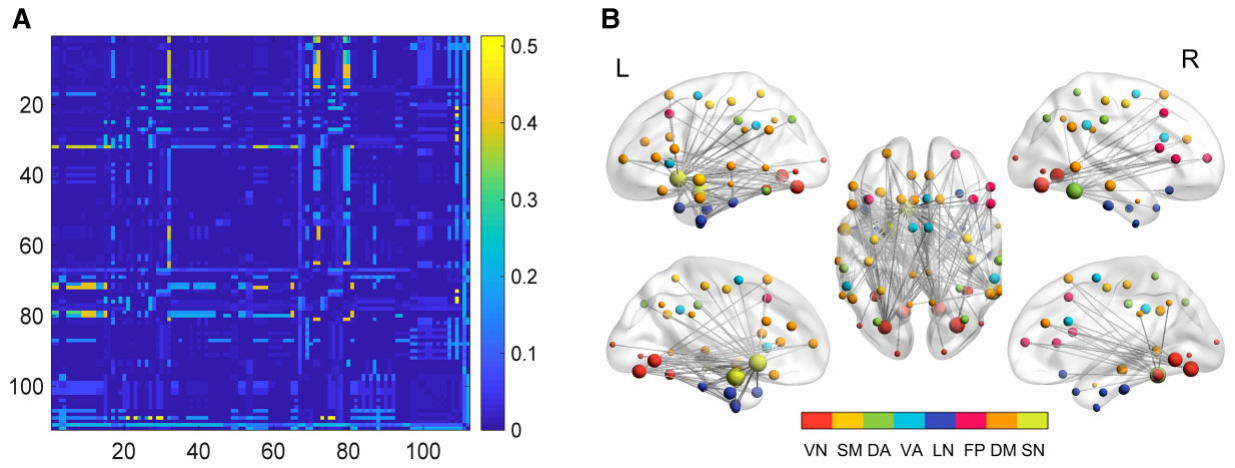
**Connection-wise contributions.** (**A**) The contribution weight of basic FCs in the classifying task. Colour bar shows the importance scale—normalized weights. (**B**) FCs ranked in the top 5% of the normalized weights. The size of the spheres reflects the region-wise contributions. The brain regions connected by the identified edges are labelled by different colours based on the network affiliations of each brain region. DA, dorsal attention; FP, frontoparietal; DM, default mode; LN, limbic network; SM, sensorimotor network; SN, subcortical network; L, left; R, right; VA, ventral attention; VN, visual network.

A total of 3356 low-order connections took part in the classifying task when we used the classifier with 10 repetitive 10-fold CV (Fig. 4A). The connections ranked in the top 5% (167 edges) of the normalized weights are shown in Fig. 4B and Supplementary Table 4. Note that the identified 167 connections mainly linked the ROIs shown in Fig. 3A. Specifically, the nucleus accumbens and amygdala (subcortical regions) and lingual gyrus (LG) and fusiform gyrus (visual network) exhibited rich connections with other ROIs.

### Network-wise contributions distinguishing POE infants from controls

**Figure 5:**
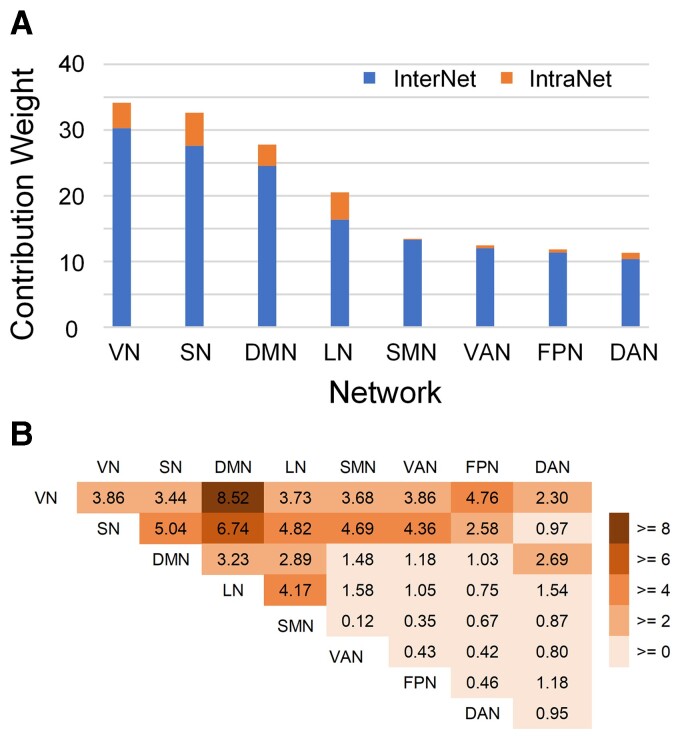
**Network-wise contribution.** (**A**) Network-level contribution in the eFC classifier to identify POE from controls. Blue bars indicate the network-level contribution of inter-network dFC, and red bars indicate that of intra-network dFC. (**B**) The contribution of each pairwise inter-network connection to the eFC classifier. DAN, dorsal attention network; DMN, default mode network; FPN, frontoparietal network; LN, limbic network; SMN, sensorimotor network; SN, subcortical network; VN, visual network; VAN, ventral attention network.

As outlined above, three network-wise contributions were calculated, including intra-network, inter-network, and pairwise inter-network indices. Fig. 5A shows both the normalized intra- and inter-network weights ranked by the whole network weights. The normalized inter-network weights are higher than the intra-network weights, suggesting that neural substrates distinguishing POE infants from controls are mostly inter-network rather than intra-network connections. Furthermore, among the inter-network contributions, the visual network exhibits the highest normalized inter-network weights, followed by the subcortical, default mode, and limbic networks, suggesting that eFC connections to these networks may be more affected by POE. The pairwise inter-network contributions (Fig. 5B) further revealed that although the visual and subcortical networks are the two most important networks (Fig. 5A), they have distinctly different patterns associated with POE; the visual-related POE-influenced FCs were mainly connected to the default mode and fronto-parietal networks, while the subcortical network showed uniformly strong influence in the FCs with the default mode, limbic, sensorimotor, and ventral attention networks.

## Discussion

The intrinsic interaction between different brain regions has been reported to be temporally nonstationary. As a result, static FC using the FC matrix obtained from the entire rs-fMRI scan^54^ may fail to capture brain functions that are only present within time scales much shorter than the entire data acquisition.^36^ Recently, edge-centric methods have been proposed capable of capturing interaction patterns between pairs of edges, which differ from the widely used static FC assessing temporal synchrony of BOLD signals among pairs of brain regions.^55,56^ The patterns of co-fluctuation revealed by eFCs could reflect the aggregate effects of communication processes between neural elements.^55–57^ Faskowitz *et al*^55^ investigated eFCs and revealed an overlapping neural system-level architecture. That is, a given brain region could be assigned to multiple brain functional networks. In particular, the greatest levels of overlap were observed in the sensorimotor and attentional networks. Jo *et al*^56^ used eFCs to improve subject idiosyncrasies and captured subject-specific features as fingerprints to characterize unique individuals. In this study, we developed an edge-centric representation of dynamic FC (dFC) and used it distinguish infants with POE from typically developing infants. Our results showed that the eFC approach outperformed the widely used static FC for distinguishing POE infants from controls (Table 2), suggesting that the neural substrates underpinning POE infants may be associated with alterations of co-fluctuation between pairs of edges instead of temporal synchrony between pairs of brain regions. Collectively, eFC has provided a new tool studying the higher-order organization and function of brain networks and serves as complementary approaches to the current prevailing node-based static FC.

Furthermore, our results indicate that the effects of POE may predominately alter inter-network instead of intra-network dFC (Fig. 4 and Fig. 5). We previously reported that long-distance and inter-network connections appear to be more vulnerable in POE infants when compared to controls.^28^ In this study, the eFC results further elucidated the differences of high-order communication in inter-network eFC. Specifically, we found that inter-network eFC with the visual, subcortical, and default mode networks were more strongly associated (larger inter-network weights, Fig. 5) with POE than that of other functional networks, which implied that the high-order interactions between them could be more affected by POE. While inter-network connections serve less specialized and complex brain functions when compared to intra-network connections,^58,59^ inter-network communications have been regarded as essential to maintain healthy executive function.^58,59^ In particular, growing evidence from adult studies^60,61^ has supported that the intrinsic neural connections between visual and default mode networks play a key role in visual mental imagery, which is the basis of a variety of high-level cognitive functions.^62^ Better visual creativity was correlated with stronger resting-state FC between the visual network, default mode, and frontoparietal networks.^61,63^ Although our studies focused on infants, it is plausible that the similar mechanisms are present at some level or are developing in infants. We found that the inter-network connectivity between the visual and DMN exhibited the largest weight, followed by visual-FPN, suggesting potential functional alterations governed by the inter-network connectivity among the three networks in POE infants. Indeed, children with POE are known to have a higher risk of visual problems, including reduced visual acuity, strabismus, and nystagmus.^64–67^ After prenatal exposure to methadone, infants show abnormal, smaller, or slower visual evoked potentials relative to controls.^68^ Furthermore, infants with POE experiencing withdrawal symptoms tend to be ‘disorganized’ soon after birth and require decreased auditory and visual stimulations.^28^ The inter-network connectional differences shown in this study may reflect this outward disorganization. We speculated that the observed abnormalities of higher order inter-network connections between visual and default mode as well as frontoparietal networks may be the neurological underpinning of higher risks of altered visual function in children with POE.

Consistent with the observed inter-network dFC features between the visual and default mode networks of POE infants, we also found that the bilateral occipital fusiform gyrus, LG, temporal occipital fusiform gyrus (TOF), and right temporal fusiform cortex were among the highest regional contributions for differentiating POE from controls (Fig. 3B). The fusiform gyrus plays an important role for high-order visual processing in children and adults, including identification and differentiation of objects,^69,70^ face recognition,^69,71,72^ processing of colour information,^73–75^ identifying words and letters from lower-level shape images,^74,76,77^ and accessing object semantic information.^74,75,78^ Furthermore, the LG has been shown to govern visual processing,^79,80^ analysis of logical conditions,^80,81^ and encoding of visual memories.^80^ Normal visual function is important for brain maturation and cognitive development. Infant’s visual performance such as attention and fixation has been implicated to predict later neurocognitive development.^82,83^ Neuroimaging features such as the flexibility of the visual network has been associated with later cognitive performance.^84^ Together, these findings may potentially explain the observed differences of visual functions and later cognitive delays in infants with POE.

The subcortical network was the second most important network distinguishing POE infants from controls (Fig. 5A). The subcortical network has been linked to a variety of functions including emotion, behaviour, memory, and olfaction.^85–87^ Therefore, the effects of POE on subcortical network and regions are likely to affect a wide range of networks in the brain. Indeed, our results showed that subcortical network exhibited a distinctly different POE-affected pattern when compared to the visual network. Aside from a relatively large weight with the default mode network, the intra-network weights were more similar with the limbic, sensorimotor, and ventral attention networks. These findings suggest that high-order co-fluctuation abnormalities with the subcortical network occurred more broadly across the whole brain.

At the brain regional level, our results showed that the left amygdala and the nucleus accumbens were the two strongest regional contributors (Fig. 3B) discriminating POE from controls, consistent with the previously reported opioid-related findings in both human and animal studies.^26,27,29,88–91^ Specifically, the amygdala plays a pivotal role in emotional processing as the integrative centre for emotions, emotional behaviour, and motivation and its abnormalities have been linked to social anxiety, obsessive and compulsive disorders, and post-traumatic stress, as well as more broadly to separation and general anxiety.^92^ It is believed to represent a core fear system in the human brain.^93^ In addition, the early development of the left amygdala may provide infants the ability to detect danger.^94^ Grewen, Salzwedel and Gao^26^ observed hyper-connectivity of left amygdala with orbital frontal cortex and hypo-connectivity of posterior thalamus with hippocampus in infants with prenatal exposures to opioids and other drugs. Salzwedel et al.^27^ also revealed common drug exposure-related connectivity disruptions within the amygdala–frontal circuits. More recently, Radhakrishnan et al.^29^ found significant differences in connectivity of an amygdala seed to several cortical regions.

The nucleus accumbens, one of the subcortical nuclei, is important in addiction^95^ as it involves in the cognitive processing of motivation, aversion, and reinforcement learning,^96–98^ as well as playing a role in processing fear and impulsivity.^99–101^ A negative effect of opioids on the nucleus accumbens have also been reported.^88–91^ The neural activity of nucleus accumbens has been found to be fine-tuned by the opioid system,^102,103^ which is formed by opioid receptors and their ligands, and the opioid peptides (prodynorphin, proenkephalin and proopiomelanocortin).^104^ Mu opioid receptors are considered the main pharmacological target of opiates and the molecular substrate of their analgesic and hedonic properties. The nucleus accumbens expresses high levels of kappa, mu and delta opioid receptors, prodynorphin and proenkephalin, thus serving as a key hub of the reward system.^102,103^ Our results showed that the nucleus accumbens possessed a strong discriminative ability when distinguishing POE infants from controls, suggesting the disruption of its high-order connections with other brain regions, which may cause the dysfunction of the reward system. Together, our findings along with previously reported results, suggest effects of POE on connectional patterns of the subcortical networks, which may explain some of the later onset of emotion and inhibition abnormalities observed in children exposed to opioids prenatally.^26,27,29,90^

### Limitations

It should be noted that several confounds should be considered in our study. Specifically, 93% of the mothers of the POE infants were smokers (versus 4% in controls), and 67% of them were positive for hepatitis C (versus 0% in controls). With respect to hepatitis C, the risk for mother-to-child transmission is very low (<5%), making it unlikely to alter brain FC in the POE infants.^105,106^ However, both morphine and nicotine are considered narcotic drugs, as they both activate the mu opioid receptor at some stage,^107^ meaning that the observed differences in functional activity in infants with POE may not be specific to opiates. Nevertheless, multiple drug use is one of the common limitations of most human studies of addiction, and rather difficult to avoid.^108^ More POE infants (6/21) than the controls (4/28) were excluded due to the presence of excessive motion during image acquisition. The presence of excessive motion during MR imaging may represent hyperactivity of POE infants, a consequence of POE. Thus, the exclusion of POE infants with increased motion during MRI might have limited our findings. The sample size is limited. Nevertheless, our results are robust via the permutation test. With the machine learning approach employed in our study, neural features capable of distinguishing POE infants from controls were revealed. However, we were unable to evaluate the direction of the differences owing to the intrinsic limitation of machine learning approaches. Finally, the lack of a long-term follow-up of the study cohort makes it difficult to determine if the observed neural substrates persist beyond infancy. Nevertheless, these aforementioned limitations are not unique considering the difficulties of study on this vulnerable population. The upcoming HEALthy Brain and Child Development study, which aims to recruit a larger sample size (>5000 infants) and a longitudinal design (>5yrs), will further improve our understanding how the type, timing, and duration of opioid exposure affect the brain and relate to later neurodevelopment.

## Supplementary Material

fcac112_Supplementary_Data

## Abbreviations

AAL =: automated anatomical labelling atlas
ACC =: accuracy
ACCU =: accumbens
AG =: angular gyrus
AMYG =: amygdala
AUC =: area under the curve
BOLD =: blood oxygenation level dependent
CV =: cross-validation
DAN =: dorsal attention network
dFC =: dynamic functional connection
DMN =: default mode network
eFC =: edge-centric functional connection
FA =: flip angle
FC =: functional connection
FD =: framewise displacement
FDR =: false discovery rate
FOpC =: frontal operculum cortex
FOrC =: frontal orbital cortex
FP =: fronto-parietal network
HCP =: Human Connectome Project
HIP =: hippocampus
IC =: insular cortex
ICA =: independent component analysis
ITGa =: inferior temporal gyrus, anterior division
ITGp =: inferior temporal gyrus, posterior division
ITGt =: inferior temporal gyrus, temporooccipital part
LASSO =: least absolute shrinkage and selection operator
LG =: lingual gyrus
LN =: limbic network
MNI =: Montreal Neurological Institute
MTGa =: middle temporal gyrus, anterior division
MTGp =: middle temporal gyrus, posterior division
MTGt =: middle temporal gyrus, temporooccipital part
OFG =: occipital fusiform gyrus
OUD =: opioid use disorder
PhGa =: parahippocampal gyrus, anterior division
PhGp =: parahippocampal gyrus, posterior division
POE =: prenatal opioid exposure
PP =: planum polare
ROC =: receiver operating characteristics
ROI =: region of interest
rs-fMRI =: resting state functional MRI
SEN =: sensitivity
SENSE =: sensitivity encoding
sFC =: static functional connectivity
SmGp =: supramarginal gyrus, posterior division
SMN =: sensorimotor network
SN =: subcortical network
SPE =: specificity
STGa =: superior temporal gyrus, anterior division
SVM =: support vector machine
TE =: echo time
TFCp =: temporal fusiform cortex, posterior division
TOF =: temporal occipital fusiform cortex
TP =: temporal pole
TR =: repetition time
VAN =: ventral attention network
VN =: visual network
WLCC =: weighted local clustering coefficients
