# Supplementary material for: Neural alterations in opioid-exposed infants revealed by edge-centric brain functional networks
Source: Brain Commun. 2022 May 5;4(3):fcac112. doi: 10.1093/braincomms/fcac112 (PMC9117006; doi:10.1093/braincomms/fcac112)
Supplement: fcac112_Supplementary_Data [file fcac112_supplementary_data.docx]

**Supplement Materials**

Neural alterations in opioid-exposed infants revealed by edge-centric brain functional networks

Weixiong Jiang^1^, Stephanie L. Merhar^2^, Zhuohao Zeng^3^, Ziliang Zhu^4^, Weiyan Yin^1^, Zhen Zhou^1^，Li Wang^1^, Lili He^5^, Jennifer Vannest^6^, Weili Lin^1,7^

1. Biomedical Research Imaging Center, University of North Carolina at Chapel Hill, Chapel Hill, North Carolina, United States

2. Perinatal Institute, Division of Neonatology, Cincinnati Children’s Hospital and University of Cincinnati Department of Pediatrics, Cincinnati OH, United States

3. East Chapel Hill High School, Chapel Hill, North Carolina, United States

4. Department of Biostatistics, University of North Carolina at Chapel Hill, Chapel Hill, North Carolina, United States

5. Department of Radiology, Cincinnati Children’s Hospital and University of Cincinnati, Cincinnati OH, United States

6. Department of Communication Sciences and Disorders, College of Allied Health Sciences, University of Cincinnati, Cincinnati OH, United States

7. Department of Radiology, University of North Carolina at Chapel Hill, Chapel Hill, North Carolina, United States

**A representative example of the eFC differences between opioid-exposed infants and controls**

In this study, we used a series of edge-centric functional connectivity (eFC) networks with different sliding windows (50–120 TRs with a step size of 10 TRs) and cluster number (100–800 clusters with a step of 100) to obtain the best classifying performance. For each combination of sliding window lengths and cluster sizes, an eFC network was obtained. Our classifying results found that the eFC network with a combination of a sliding window length of 50s and a cluster number of 100 appeared the most frequently in the 10 times 10-fold cross validation. Therefore, we compared the eFC networks between the opioid-exposed infants and controls using the above-mentioned combination and the significant eFCs (without correcting for multiple comparisons) were shown in Table 4. Importantly, these results show that the eFC strengths are mostly weaker in the opioid-exposed infants (93.94%) than that of controls.

**Supplementary Table 1**. The weight of the brain regions and their corresponding abbreviations (Abbr.). L: left; R: right.

| Regions | Abbr. | Weight | Regions | Abbr. | Weight |
| --- | --- | --- | --- | --- | --- |
| Amygdala (L) | AMYG_L | 34.45 | Juxtapositional Lobule Cortex (R) | JpL_R | 3.56 |
| Accumbens (L) | ACCU_L | 31.51 | Temporal Fusiform Cortex, posterior division (L) | TFCp_L | 3.56 |
| Inferior Temporal Gyrus, temporooccipital part (R) | ITGt_R | 24.71 | Precentral Gyrus (L) | PrG_L | 3.52 |
| Occipital Fusiform Gyrus (R) | OFG_R | 19.36 | Precentral Gyrus (R) | PrG_R | 3.52 |
| Lingual Gyrus (R) | LG_R | 15.23 | Postcentral Gyrus (R) | PoG_R | 3.52 |
| Accumbens (R) | ACCU_R | 13.23 | Inferior Temporal Gyrus, posterior division (L) | ITGp_L | 3.52 |
| Occipital Fusiform Gyrus (L) | OFG_L | 12.54 | Postcentral Gyrus (L) | PoG_L | 3.51 |
| Lingual Gyrus (L) | LG_L | 12.19 | Superior Temporal Gyrus, posterior division (R) | STGp_R | 3.51 |
| Superior Temporal Gyrus, anterior division (L) | STGa_L | 9.04 | Frontal Operculum Cortex (R) | FOpC_R | 3.36 |
| Hippocampus (L) | HIP_L | 8.91 | Superior Temporal Gyrus, posterior division (L) | STGp_L | 3.35 |
| Parahippocampal Gyrus, anterior division (L) | PhGa_L | 8.78 | Temporal Fusiform Cortex, anterior division (L) | TFCa_L | 3.28 |
| Parahippocampal Gyrus, posterior division (L) | PhGp_L | 8.07 | Frontal Orbital Cortex (R) | FOrC_R | 3.20 |
| Temporal Occipital Fusiform Cortex (R) | TOF_R | 6.85 | Cuneal Cortex (L) | CC_L | 3.18 |
| Planum Polare (L) | PP_L | 6.75 | Cuneal Cortex (R) | CC_R | 3.18 |
| Middle Temporal Gyrus, posterior division (R) | MTGp_R | 6.27 | Supracalcarine Cortex (L) | ScC_L | 3.15 |
| Inferior Temporal Gyrus, anterior division (L) | ITGa_L | 6.26 | Supracalcarine Cortex (R) | ScC_R | 3.15 |
| Middle Temporal Gyrus, temporooccipital part (R) | MTGt_R | 6.24 | Intracalcarine Cortex (L) | IcC_L | 3.15 |
| Temporal Pole (L) | TP_L | 6.02 | Intracalcarine Cortex (R) | IcC_R | 3.15 |
| Middle Temporal Gyrus, anterior division (L) | MTGa_L | 5.36 | Parietal Operculum Cortex (R) | POC_R | 3.10 |
| Temporal Occipital Fusiform Cortex (L) | TOF_L | 5.22 | Heschls Gyrus (includes H1 and H2) (R) | HesG_R | 3.02 |
| Frontal Orbital Cortex (L) | FOrC_L | 5.20 | Planum Temporale (R) | PT_R | 3.02 |
| Frontal Operculum Cortex (L) | FOpC_L | 5.06 | Parahippocampal Gyrus, anterior division (R) | PhGa_R | 2.97 |
| Angular Gyrus (R) | AG_R | 5.01 | Central Opercular Cortex (R) | COC_R | 2.96 |
| Supramarginal Gyrus, posterior division (R) | SmGp_R | 5.01 | Lateral Occipital Cortex, superior division (L) | LOCs_L | 2.88 |
| Insular Cortex (R) | IC_R | 4.74 | Lateral Occipital Cortex, superior division (R) | LOCs_R | 2.88 |
| Inferior Temporal Gyrus, posterior division (R) | ITGp_R | 4.62 | Frontal Medial Cortex (L) | FMC_L | 2.78 |
| Temporal Fusiform Cortex, posterior division (R) | TFCp_R | 4.59 | Frontal Medial Cortex (R) | FMC_R | 2.78 |
| Inferior Frontal Gyrus, pars opercularis (L) | IFGpo_L | 4.50 | Superior Parietal Lobule (L) | SPL_L | 2.75 |
| Inferior Frontal Gyrus, pars triangularis (L) | IFGpt_L | 4.49 | Inferior Temporal Gyrus, temporooccipital part (L) | ITGt_L | 2.67 |
| Brain-Stem (L) | BStem_L | 4.44 | Hippocampus (R) | HIP_R | 2.66 |
| Brain-Stem (R) | BStem_R | 4.44 | Amygdala (R) | AMYG_R | 2.66 |
| Planum Polare (R) | PP_R | 4.42 | Superior Parietal Lobule (R) | SPL_R | 2.54 |
| Inferior Frontal Gyrus, pars triangularis (R) | IFGpt_R | 4.34 | Parietal Operculum Cortex (L) | POC_L | 2.54 |
| Inferior Frontal Gyrus, pars opercularis (R) | IFGpo_R | 4.34 | Planum Temporale (L) | PT_L | 2.52 |
| Insular Cortex (L) | IC_L | 4.29 | Temporal Pole (R) | TP_R | 2.49 |
| Paracingulate Gyrus (L) | PcG_L | 4.22 | Heschls Gyrus (includes H1 and H2) (L) | HesG_L | 2.35 |
| Paracingulate Gyrus (R) | PcG_R | 4.22 | Central Opercular Cortex (L) | COC_L | 2.31 |
| Cingulate Gyrus, anterior division (L) | CGa_L | 4.22 | Putamen (R) | PUT_R | 2.31 |
| Cingulate Gyrus, anterior division (R) | CGa_R | 4.22 | Parahippocampal Gyrus, posterior division (R) | PhGp_R | 2.27 |
| Frontal Pole (L) | FP_L | 4.20 | Pallidum (R) | PAL_R | 2.25 |
| Frontal Pole (R) | FP_R | 4.20 | Middle Temporal Gyrus, temporooccipital part (L) | MTGt_L | 2.05 |
| Caudate (R) | CAU_R | 4.19 | Temporal Fusiform Cortex, anterior division (R) | TFCa_R | 2.00 |
| Caudate (L) | CAU_L | 4.18 | Pallidum (L) | PAL_L | 1.87 |
| Middle Frontal Gyrus (L) | MFG_L | 4.14 | Putamen (L) | PUT_L | 1.87 |
| Middle Frontal Gyrus (R) | MFG_R | 4.13 | Cingulate Gyrus, posterior division (L) | CGp_L | 1.82 |
| Superior Frontal Gyrus (L) | SFG_L | 4.13 | Cingulate Gyrus, posterior division (R) | CGp_R | 1.82 |
| Superior Frontal Gyrus (R) | SFG_R | 4.13 | Inferior Temporal Gyrus, anterior division (R) | ITGa_R | 1.62 |
| Supramarginal Gyrus, anterior division (R) | SmGa_R | 3.91 | Precuneous Cortex (L) | PcC_L | 1.61 |
| Thalamus (L) | THA_L | 3.91 | Precuneous Cortex (R) | PcC_R | 1.61 |
| Thalamus (R) | THA_R | 3.91 | Middle Temporal Gyrus, posterior division (L) | MTGp_L | 1.31 |
| Supramarginal Gyrus, anterior division (L) | SmGa_L | 3.83 | Occipital Pole (L) | OcP_L | 1.19 |
| Supramarginal Gyrus, posterior division (L) | SmGp_L | 3.83 | Occipital Pole (R) | OcP_R | 1.19 |
| Angular Gyrus (L) | AG_L | 3.83 | Lateral Occipital Cortex, inferior division (L) | LOCi_L | 1.14 |
| Subcallosal Cortex (L) | SubC_L | 3.62 | Lateral Occipital Cortex, inferior division (R) | LOCi_R | 1.09 |
| Subcallosal Cortex (R) | SubC_R | 3.62 | Middle Temporal Gyrus, anterior division (R) | MTGa_R | 0.90 |
| Juxtapositional Lobule Cortex (L) | JpL_L | 3.56 | Superior Temporal Gyrus, anterior division (R) | STGa_R | 0.85 |
|  |  |  |  |  |  |

**Supplementary Table 2**. Comparisons of edge-centric functional connectivity (window 50 s and cluster 100) between opioid-exposed infants and controls.

| eFC (index*) | T | P | eFC (index) | T | P |
| --- | --- | --- | --- | --- | --- |
| 202 | -3.22 | 0.0028 | 2573 | -3.89 | 0.0004 |
| 217 | -3.00 | 0.0050 | 2582 | -3.04 | 0.0044 |
| 218 | -2.90 | 0.0064 | 2585 | -2.76 | 0.0091 |
| 225 | -3.05 | 0.0043 | 2603 | -2.84 | 0.0075 |
| 233 | -3.17 | 0.0031 | 2604 | -3.54 | 0.0012 |
| 236 | -3.42 | 0.0016 | 2641 | -2.78 | 0.0087 |
| 542 | -3.21 | 0.0029 | 2672 | -3.63 | 0.0009 |
| 547 | -2.78 | 0.0087 | 3070 | -2.96 | 0.0055 |
| 802 | 2.74 | 0.0095 | 3073 | -2.76 | 0.0090 |
| 806 | 3.00 | 0.0050 | 3075 | -3.10 | 0.0038 |
| 1148 | -3.48 | 0.0014 | 3088 | -2.81 | 0.0081 |
| 1209 | -3.27 | 0.0024 | 3102 | -2.90 | 0.0065 |
| 1234 | -3.03 | 0.0045 | 3130 | -3.98 | 0.0003 |
| 1235 | -2.79 | 0.0084 | 3154 | -2.75 | 0.0093 |
| 1294 | -3.03 | 0.0046 | 3410 | -2.96 | 0.0055 |
| 1537 | -3.18 | 0.0031 | 3469 | 2.78 | 0.0087 |
| 1823 | -2.84 | 0.0075 | 3520 | -3.05 | 0.0044 |
| 1845 | -3.17 | 0.0032 | 3649 | -3.47 | 0.0014 |
| 1873 | -2.86 | 0.0071 | 3859 | -2.87 | 0.0069 |
| 1932 | -3.22 | 0.0027 | 3937 | -2.80 | 0.0084 |
| 2040 | -3.14 | 0.0035 | 3960 | -2.78 | 0.0087 |
| 2045 | -2.72 | 0.0100 | 4194 | -3.26 | 0.0025 |
| 2054 | -3.14 | 0.0034 | 4232 | -2.81 | 0.0080 |
| 2063 | -3.22 | 0.0028 | 4256 | -2.79 | 0.0086 |
| 2138 | -3.19 | 0.0030 | 4258 | -3.03 | 0.0046 |
| 2212 | -2.75 | 0.0094 | 4266 | -3.06 | 0.0042 |
| 2357 | -2.74 | 0.0096 | 4320 | -2.73 | 0.0098 |
| 2387 | 2.87 | 0.0070 | 4500 | -3.35 | 0.0019 |
| 2428 | -2.90 | 0.0063 | 4576 | -2.73 | 0.0098 |
| 2540 | -2.87 | 0.0068 | 4656 | -2.86 | 0.0072 |
| 2553 | -3.70 | 0.0007 | 4797 | -2.86 | 0.0072 |
| 2566 | -2.95 | 0.0056 | 4845 | -3.06 | 0.0042 |
| 2567 | -2.97 | 0.0054 | 4901 | -2.74 | 0.0097 |

*Index indicates the numeration of edge-centric functional

connectivity (eFC).

**Supplementary Table 3**. The network affiliation of each region of interest (ROI). VN: visual network; SMN: sensorimotor network; DAN: dorsal attention network; VAN: ventral attention network; LN: limbic network; FPN: frontoparietal network; DMN: default mode network: SN: subcortical network. L: left; R: right. The abbreviation of each ROI is shown in Table S1.

| No. | ROI | VN | SMN | DAN | VAN | LN | FPN | DMN | SN | No. | ROI | VN | SMN | DAN | VAN | LN | FPN | DMN | SN |
| --- | --- | --- | --- | --- | --- | --- | --- | --- | --- | --- | --- | --- | --- | --- | --- | --- | --- | --- | --- |
| 1 | FP_L | 0 | 0 | 0 | 0 | 0 | 0.4958 | 0.5042 | 0 | 57 | CGa_L | 0 | 0 | 0 | 0.4527 | 0 | 0 | 0.5473 | 0 |
| 2 | FP_R | 0 | 0 | 0 | 0 | 0 | 0.6478 | 0.3522 | 0 | 58 | CGa_R | 0 | 0 | 0 | 0.5378 | 0 | 0 | 0.4622 | 0 |
| 3 | IC_L | 0 | 0 | 0 | 1 | 0 | 0 | 0 | 0 | 59 | CGp_L | 0 | 0 | 0 | 0 | 0 | 0 | 1 | 0 |
| 4 | IC_R | 0 | 0 | 0 | 0.5662 | 0 | 0 | 0.4338 | 0 | 60 | CGp_R | 0 | 0 | 0 | 0 | 0 | 0 | 1 | 0 |
| 5 | SFG_L | 0 | 0 | 0 | 0 | 0 | 0 | 1 | 0 | 61 | PcC_L | 0 | 0 | 0 | 0 | 0 | 0 | 1 | 0 |
| 6 | SFG_R | 0 | 0 | 0 | 0 | 0 | 0.4492 | 0.5508 | 0 | 62 | PcC_R | 0 | 0 | 0.4104 | 0 | 0 | 0 | 0.5896 | 0 |
| 7 | MFG_L | 0 | 0 | 0 | 0 | 0 | 0.5588 | 0.4412 | 0 | 63 | CC_L | 1 | 0 | 0 | 0 | 0 | 0 | 0 | 0 |
| 8 | MFG_R | 0 | 0 | 0 | 0 | 0 | 1 | 0 | 0 | 64 | CC_R | 1 | 0 | 0 | 0 | 0 | 0 | 0 | 0 |
| 9 | IFGpt_L | 0 | 0 | 0 | 0 | 0 | 0.4708 | 0.5292 | 0 | 65 | FOrC_L | 0 | 0 | 0 | 0 | 0.4842 | 0 | 0.5158 | 0 |
| 10 | IFGpt_R | 0 | 0 | 0 | 0 | 0 | 0.5584 | 0.4416 | 0 | 66 | FOrC_R | 0 | 0 | 0 | 0 | 0.6005 | 0 | 0.3995 | 0 |
| 11 | IFGpo_L | 0 | 0 | 0 | 0 | 0 | 0.3717 | 0.6283 | 0 | 67 | PhGa_L | 0 | 0 | 0 | 0 | 1 | 0 | 0 | 0 |
| 12 | IFGpo_R | 0 | 0 | 0 | 0 | 0 | 1 | 0 | 0 | 68 | PhGa_R | 0 | 0 | 0 | 0 | 1 | 0 | 0 | 0 |
| 13 | PrG_L | 0 | 1 | 0 | 0 | 0 | 0 | 0 | 0 | 69 | PhGp_L | 0.5756 | 0 | 0 | 0 | 0 | 0 | 0.4244 | 0 |
| 14 | PrG_R | 0 | 1 | 0 | 0 | 0 | 0 | 0 | 0 | 70 | PhGp_R | 1 | 0 | 0 | 0 | 0 | 0 | 0 | 0 |
| 15 | TP_L | 0 | 0 | 0 | 0 | 0.6707 | 0 | 0.3293 | 0 | 71 | LG_L | 1 | 0 | 0 | 0 | 0 | 0 | 0 | 0 |
| 16 | TP_R | 0 | 0 | 0 | 0 | 1 | 0 | 0 | 0 | 72 | LG_R | 1 | 0 | 0 | 0 | 0 | 0 | 0 | 0 |
| 17 | STGa_L | 0 | 0.5092 | 0 | 0 | 0 | 0 | 0.4908 | 0 | 73 | TFCa_L | 0.2903 | 0 | 0 | 0 | 0.7097 | 0 | 0 | 0 |
| 18 | STGa_R | 0 | 0.4682 | 0 | 0 | 0 | 0 | 0.5318 | 0 | 74 | TFCa_R | 0.3211 | 0 | 0 | 0 | 0.6789 | 0 | 0 | 0 |
| 19 | STGp_L | 0 | 0.4711 | 0 | 0 | 0 | 0 | 0.5289 | 0 | 75 | TFCp_L | 0.4309 | 0 | 0 | 0 | 0.5691 | 0 | 0 | 0 |
| 20 | STGp_R | 0 | 1 | 0 | 0 | 0 | 0 | 0 | 0 | 76 | TFCp_R | 0.4948 | 0 | 0 | 0 | 0.5052 | 0 | 0 | 0 |
| 21 | MTGa_L | 0 | 0 | 0 | 0 | 0 | 0 | 1 | 0 | 77 | TOF_L | 1 | 0 | 0 | 0 | 0 | 0 | 0 | 0 |
| 22 | MTGa_R | 0 | 0 | 0 | 0 | 0 | 0 | 1 | 0 | 78 | TOF_R | 1 | 0 | 0 | 0 | 0 | 0 | 0 | 0 |
| 23 | MTGp_L | 0 | 0 | 0 | 0 | 0 | 0 | 1 | 0 | 79 | OFG_L | 1 | 0 | 0 | 0 | 0 | 0 | 0 | 0 |
| 24 | MTGp_R | 0 | 0 | 0 | 0 | 0 | 0 | 1 | 0 | 80 | OFG_R | 1 | 0 | 0 | 0 | 0 | 0 | 0 | 0 |
| 25 | MTGt_L | 0 | 0 | 0 | 0 | 0 | 0 | 1 | 0 | 81 | FOpC_L | 0 | 0 | 0 | 1 | 0 | 0 | 0 | 0 |
| 26 | MTGt_R | 0 | 0 | 0 | 0.4979 | 0 | 0 | 0.5021 | 0 | 82 | FOpC_R | 0 | 0 | 0 | 1 | 0 | 0 | 0 | 0 |
| 27 | ITGa_L | 0 | 0 | 0 | 0 | 1 | 0 | 0 | 0 | 83 | COC_L | 0 | 0.6314 | 0 | 0.3686 | 0 | 0 | 0 | 0 |
| 28 | ITGa_R | 0 | 0 | 0 | 0 | 1 | 0 | 0 | 0 | 84 | COC_R | 0 | 0.6154 | 0 | 0.3846 | 0 | 0 | 0 | 0 |
| 29 | ITGp_L | 0 | 0 | 0 | 0 | 1 | 0 | 0 | 0 | 85 | POC_L | 0 | 0.5893 | 0 | 0.4107 | 0 | 0 | 0 | 0 |
| 30 | ITGp_R | 0 | 0 | 0 | 0 | 1 | 0 | 0 | 0 | 86 | POC_R | 0 | 0.581 | 0 | 0.419 | 0 | 0 | 0 | 0 |
| 31 | ITGt_L | 0 | 0 | 0.5829 | 0 | 0 | 0.4171 | 0 | 0 | 87 | PP_L | 0.2582 | 0.5041 | 0 | 0.2377 | 0 | 0 | 0 | 0 |
| 32 | ITGt_R | 0 | 0 | 1 | 0 | 0 | 0 | 0 | 0 | 88 | PP_R | 0.2563 | 0.7437 | 0 | 0 | 0 | 0 | 0 | 0 |
| 33 | PoG_L | 0 | 1 | 0 | 0 | 0 | 0 | 0 | 0 | 89 | HesG_L | 0 | 1 | 0 | 0 | 0 | 0 | 0 | 0 |
| 34 | PoG_R | 0 | 1 | 0 | 0 | 0 | 0 | 0 | 0 | 90 | HesG_R | 0.2409 | 0.7591 | 0 | 0 | 0 | 0 | 0 | 0 |
| 35 | SPL_L | 0 | 0.6612 | 0.3388 | 0 | 0 | 0 | 0 | 0 | 91 | PT_L | 0 | 1 | 0 | 0 | 0 | 0 | 0 | 0 |
| 36 | SPL_R | 0 | 0.4557 | 0.5443 | 0 | 0 | 0 | 0 | 0 | 92 | PT_R | 0.4235 | 0.5765 | 0 | 0 | 0 | 0 | 0 | 0 |
| 37 | SmGa_L | 0 | 0 | 0.5557 | 0.4443 | 0 | 0 | 0 | 0 | 93 | ScC_L | 1 | 0 | 0 | 0 | 0 | 0 | 0 | 0 |
| 38 | SmGa_R | 0 | 0 | 0.5357 | 0.4643 | 0 | 0 | 0 | 0 | 94 | ScC_R | 1 | 0 | 0 | 0 | 0 | 0 | 0 | 0 |
| 39 | SmGp_L | 0 | 0 | 0 | 0.5561 | 0 | 0.4439 | 0 | 0 | 95 | OcP_L | 1 | 0 | 0 | 0 | 0 | 0 | 0 | 0 |
| 40 | SmGp_R | 0 | 0 | 0 | 0.5417 | 0 | 0.4583 | 0 | 0 | 96 | OcP_R | 1 | 0 | 0 | 0 | 0 | 0 | 0 | 0 |
| 41 | AG_L | 0 | 0 | 0 | 0 | 0 | 0.3171 | 0.6829 | 0 | 97 | BStem_L | 0 | 0 | 0 | 0 | 0 | 0 | 0 | 1 |
| 42 | AG_R | 0 | 0 | 0 | 0 | 0 | 0.4263 | 0.5737 | 0 | 98 | BStem_R | 0 | 0 | 0 | 0 | 0 | 0 | 0 | 1 |
| 43 | LOCs_L | 0 | 0 | 0.5947 | 0 | 0 | 0 | 0.4053 | 0 | 99 | THA_L | 0 | 0 | 0 | 0 | 0 | 0 | 0 | 1 |
| 44 | LOCs_R | 0 | 0 | 1 | 0 | 0 | 0 | 0 | 0 | 100 | THA_R | 0 | 0 | 0 | 0 | 0 | 0 | 0 | 1 |
| 45 | LOCi_L | 1 | 0 | 0 | 0 | 0 | 0 | 0 | 0 | 101 | CAU_L | 0 | 0 | 0 | 0 | 0 | 0 | 0 | 1 |
| 46 | LOCi_R | 1 | 0 | 0 | 0 | 0 | 0 | 0 | 0 | 102 | CAU_R | 0 | 0 | 0 | 0 | 0 | 0 | 0 | 1 |
| 47 | IcC_L | 1 | 0 | 0 | 0 | 0 | 0 | 0 | 0 | 103 | PUT_L | 0 | 0 | 0 | 0 | 0 | 0 | 0 | 1 |
| 48 | IcC_R | 1 | 0 | 0 | 0 | 0 | 0 | 0 | 0 | 104 | PUT_R | 0 | 0 | 0 | 0 | 0 | 0 | 0 | 1 |
| 49 | FMC_L | 0 | 0 | 0 | 0 | 0.564 | 0 | 0.436 | 0 | 105 | PAL_L | 0 | 0 | 0 | 0 | 0 | 0 | 0 | 1 |
| 50 | FMC_R | 0 | 0 | 0 | 0 | 0.5758 | 0 | 0.4242 | 0 | 106 | PAL_R | 0 | 0 | 0 | 0 | 0 | 0 | 0 | 1 |
| 51 | JpL_L | 0 | 0 | 0 | 1 | 0 | 0 | 0 | 0 | 107 | HIP_L | 0 | 0 | 0 | 0 | 0 | 0 | 0 | 1 |
| 52 | JpL_R | 0 | 0 | 0 | 1 | 0 | 0 | 0 | 0 | 108 | HIP_R | 0 | 0 | 0 | 0 | 0 | 0 | 0 | 1 |
| 53 | SubC_L | 0 | 0 | 0 | 0 | 1 | 0 | 0 | 0 | 109 | AMYG_L | 0 | 0 | 0 | 0 | 0 | 0 | 0 | 1 |
| 54 | SubC_R | 0 | 0 | 0 | 0 | 0.6149 | 0 | 0.3851 | 0 | 110 | AMYG_R | 0 | 0 | 0 | 0 | 0 | 0 | 0 | 1 |
| 55 | PcG_L | 0 | 0 | 0 | 0 | 0 | 0 | 1 | 0 | 111 | ACCU_L | 0 | 0 | 0 | 0 | 0 | 0 | 0 | 1 |
| 56 | PcG_R | 0 | 0 | 0 | 0 | 0 | 0 | 1 | 0 | 112 | ACCU_R | 0 | 0 | 0 | 0 | 0 | 0 | 0 | 1 |
|  |  |  |  |  |  |  |  |  |  |  |  |  |  |  |  |  |  |  |  |

**Supplementary Table 4**. The most discriminative connections with normalized weight ranked in the top 5%. FC: functional connectivity. L: left; R: right. The abbreviation of each ROI is shown in Table S1.

| FC | Weight | FC | Weight | FC | Weight | FC | Weight | FC | Weight |
| --- | --- | --- | --- | --- | --- | --- | --- | --- | --- |
| FP_L/ITGt_R | -0.3703 | IFGpt_R/LG_R | -0.3977 | STGa_L/ITGt_L | 0.2300 | ITGa_L/AMYG_L | -0.5132 | JpL_L/ACCU_L | 0.2224 |
| FP_L/LG_L | 0.3182 | IFGpt_R/OFG_L | -0.3977 | STGa_L/ITGt_R | 0.2300 | ITGa_L/ACCU_L | 0.2224 | JpL_R/ACCU_L | 0.2224 |
| FP_L/LG_R | -0.3977 | IFGpt_R/OFG_R | -0.3977 | STGa_L/TFCp_R | 0.2300 | ITGa_R/ACCU_L | 0.2224 | PcG_L/LG_R | -0.3977 |
| FP_L/OFG_L | 0.3182 | IFGpt_R/ACCU_L | 0.2224 | STGp_L/ITGp_L | 0.2300 | ITGp_L/AMYG_L | -0.5132 | PcG_R/LG_R | -0.3977 |
| FP_L/ACCU_L | 0.2224 | IFGpo_L/ITGt_R | -0.3703 | STGp_L/ITGp_R | 0.2300 | ITGp_L/ACCU_L | 0.2224 | CGa_L/LG_R | -0.3977 |
| FP_R/ITGt_R | -0.3703 | IFGpo_L/LG_L | -0.3977 | STGp_L/ITGt_R | 0.2300 | ITGp_R/AMYG_L | -0.5132 | CGa_R/LG_R | -0.3977 |
| FP_R/LG_L | 0.3182 | IFGpo_L/LG_R | -0.3977 | STGp_L/TFCp_L | 0.2300 | ITGp_R/ACCU_L | 0.2224 | FOrC_L/LG_L | -0.3977 |
| FP_R/LG_R | -0.3977 | IFGpo_L/OFG_L | -0.3977 | STGp_L/TFCp_R | 0.2300 | ITGt_L/ACCU_L | 0.2224 | FOrC_L/LG_R | -0.3977 |
| FP_R/OFG_L | 0.3182 | IFGpo_L/OFG_R | -0.3977 | MTGa_L/MTGt_L | 0.2300 | ITGt_R/PcG_L | -0.3703 | FOrC_L/OFG_L | -0.3977 |
| FP_R/ACCU_L | 0.2224 | IFGpo_L/ACCU_L | 0.2224 | MTGa_L/ITGp_L | 0.2300 | ITGt_R/PcG_R | -0.3703 | FOrC_L/OFG_R | -0.3977 |
| SFG_L/ITGt_R | -0.3703 | IFGpo_R/ITGt_R | -0.3703 | MTGa_L/ITGp_R | 0.2300 | ITGt_R/CGa_L | -0.3703 | PhGa_L/ACCU_L | 0.2224 |
| SFG_L/LG_R | -0.3977 | IFGpo_R/LG_L | -0.3977 | MTGa_L/ITGt_L | 0.2300 | ITGt_R/CGa_R | -0.3703 | PhGa_R/ACCU_L | 0.2224 |
| SFG_L/OFG_L | 0.3182 | IFGpo_R/LG_R | -0.3977 | MTGa_L/ITGt_R | 0.2300 | ITGt_R/CGp_L | -0.2044 | LG_L/FOpC_L | -0.3977 |
| SFG_L/ACCU_L | 0.2224 | IFGpo_R/OFG_L | -0.3977 | MTGa_L/TFCp_L | 0.2300 | ITGt_R/CGp_R | -0.2044 | LG_L/ACCU_L | 0.2224 |
| SFG_R/ITGt_R | -0.3703 | IFGpo_R/OFG_R | -0.3977 | MTGa_L/TFCp_R | 0.2300 | ITGt_R/PcC_L | -0.2044 | LG_R/FOpC_L | -0.3977 |
| SFG_R/LG_R | -0.3977 | IFGpo_R/ACCU_L | 0.2224 | MTGa_L/TOF_L | 0.2300 | ITGt_R/PcC_R | -0.2044 | LG_R/ACCU_L | 0.2224 |
| SFG_R/OFG_L | 0.3182 | PrG_L/ACCU_L | 0.2224 | MTGa_L/TOF_R | 0.2300 | ITGt_R/FOrC_L | -0.3703 | TFCa_L/AMYG_L | -0.5132 |
| SFG_R/ACCU_L | 0.2224 | PrG_R/ACCU_L | 0.2224 | MTGa_L/AMYG_L | -0.5132 | ITGt_R/FOrC_R | -0.3703 | TFCa_L/ACCU_L | 0.2224 |
| MFG_L/ITGt_R | -0.3703 | TP_L/MTGt_L | 0.2300 | MTGa_L/ACCU_L | 0.2224 | ITGt_R/FOpC_L | -0.3703 | TFCa_R/ACCU_L | 0.2224 |
| MFG_L/LG_R | -0.3977 | TP_L/ITGp_L | 0.2300 | MTGa_R/ACCU_L | 0.2224 | ITGt_R/ACCU_L | 0.2224 | TFCp_L/AMYG_L | -0.5132 |
| MFG_L/OFG_L | 0.3182 | TP_L/ITGp_R | 0.2300 | MTGp_L/AMYG_L | -0.5132 | PoG_L/ACCU_L | 0.2224 | TFCp_L/ACCU_L | 0.2224 |
| MFG_L/ACCU_L | 0.2224 | TP_L/ITGt_L | 0.2300 | MTGp_L/ACCU_L | 0.2224 | PoG_R/ACCU_L | 0.2224 | TFCp_R/AMYG_L | -0.5132 |
| MFG_R/ITGt_R | -0.3703 | TP_L/ITGt_R | -0.3703 | MTGp_R/ACCU_L | 0.2224 | SPL_L/ACCU_L | 0.2224 | TFCp_R/ACCU_L | 0.2224 |
| MFG_R/LG_R | -0.3977 | TP_L/LG_L | -0.3977 | MTGt_L/ITGa_L | 0.2300 | SPL_R/ACCU_L | 0.2224 | TOF_L/ACCU_L | 0.2224 |
| MFG_R/OFG_L | 0.3182 | TP_L/LG_R | -0.3977 | MTGt_L/ACCU_L | 0.2224 | SmGa_L/ACCU_L | 0.2224 | TOF_R/ACCU_L | 0.2224 |
| MFG_R/ACCU_L | 0.2224 | TP_L/TFCp_L | 0.2300 | MTGt_R/ACCU_L | 0.2224 | SmGa_R/ACCU_L | 0.2224 | OFG_L/FOpC_L | -0.3977 |
| IFGpt_L/ITGt_R | -0.3703 | TP_L/TFCp_R | 0.2300 | ITGa_L/ITGp_L | 0.2300 | SmGp_L/ACCU_L | 0.2224 | OFG_L/ACCU_L | 0.2224 |
| IFGpt_L/LG_L | -0.3977 | TP_L/OFG_L | -0.3977 | ITGa_L/ITGp_R | 0.2300 | SmGp_R/ACCU_L | 0.2224 | OFG_R/FOpC_L | -0.3977 |
| IFGpt_L/LG_R | -0.3977 | TP_L/OFG_R | -0.3977 | ITGa_L/ITGt_L | 0.2300 | AG_L/ACCU_L | 0.2224 | OFG_R/ACCU_L | 0.2224 |
| IFGpt_L/OFG_L | -0.3977 | TP_L/ACCU_L | 0.2224 | ITGa_L/ITGt_R | 0.2300 | AG_R/ACCU_L | 0.2224 | OcP_L/ACCU_L | 0.2224 |
| IFGpt_L/OFG_R | -0.3977 | TP_R/ITGt_R | -0.3703 | ITGa_L/TFCp_L | 0.2300 | LOCs_L/ACCU_L | 0.2224 | OcP_R/ACCU_L | 0.2224 |
| IFGpt_L/ACCU_L | 0.2224 | TP_R/ACCU_L | 0.2224 | ITGa_L/TFCp_R | 0.2300 | LOCs_R/ACCU_L | 0.2224 |  |  |
| IFGpt_R/ITGt_R | -0.3703 | STGa_L/MTGt_L | 0.2300 | ITGa_L/TOF_L | 0.2300 | LOCi_L/ACCU_L | 0.2224 |  |  |
| IFGpt_R/LG_L | -0.3977 | STGa_L/ITGp_R | 0.2300 | ITGa_L/TOF_R | 0.2300 | LOCi_R/ACCU_L | 0.2224 |  |  |
|  |  |  |  |  |  |  |  |  |  |


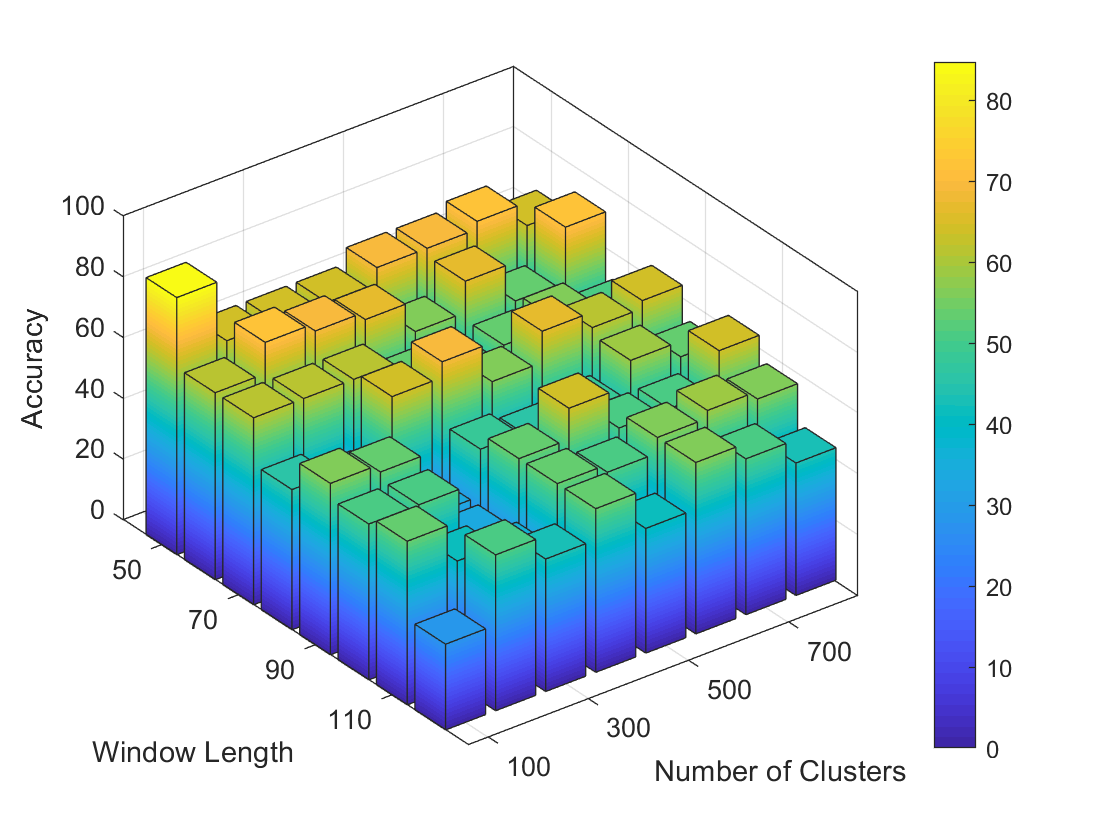


**Supplementary Figure 1**. The effect of window length and number of clusters on the classification accuracy based on edge-centric FC using one time ten-fold validation. The color bar shows classifying accuracy.
